# Engineering elastic sealants based on gelatin and elastin‐like polypeptides for endovascular anastomosis

**DOI:** 10.1002/btm2.10240

**Published:** 2021-08-10

**Authors:** Gokberk Unal, Jesse Jones, Sevana Baghdasarian, Naoki Kaneko, Ehsan Shirzaei Sani, Sohyung Lee, Shima Gholizadeh, Satoshi Tateshima, Nasim Annabi

**Affiliations:** ^1^ Department of Chemical and Biomolecular Engineering University of California Los Angeles California USA; ^2^ Division of Interventional Neuroradiology David Geffen School of Medicine at UCLA Los Angeles California USA; ^3^ Department of Neurosurgery The University of Alabama Birmingham Alabama USA

**Keywords:** cerebrovascular ischemia, crosslinking, ELP, endovascular anastomosis, GelMA, hydrogel, surgical glue, visible light

## Abstract

Cerebrovascular ischemia from intracranial atherosclerosis remains difficult to treat. Although current revascularization procedures, including intraluminal stents and extracranial to intracranial bypass, have shown some benefit, they suffer from perioperative and postoperative morbidity. To address these limitations, here we developed a novel approach that involves gluing of arteries and subsequent transmural anastomosis from the healthy donor into the ischemic recipient. This approach required an elastic vascular sealant with distinct mechanical properties and adhesion to facilitate anastomosis. We engineered two hydrogel‐based glues: an elastic composite hydrogel based on methacryloyl elastin‐like polypeptide (mELP) combined with gelatin methacryloyl (GelMA) and a stiff glue based on pure GelMA. Two formulations with distinct mechanical characteristics were necessary to achieve stable anastomosis. The elastic GelMA/mELP composite glue attained desirable mechanical properties (elastic modulus: 288 ± 19 kPa, extensibility: 34.5 ± 13.4%) and adhesion (shear strength: 26.7 ± 5.4 kPa) to the blood vessel, while the pure GelMA glue exhibited superior adhesion (shear strength: 49.4 ± 7.0 kPa) at the cost of increased stiffness (elastic modulus: 581 ± 51 kPa) and reduced extensibility (13.6 ± 2.5%). The in vitro biocompatibility tests confirmed that the glues were not cytotoxic and were biodegradable. In addition, an ex vivo porcine anastomosis model showed high arterial burst pressure resistance of 34.0 ± 7.5 kPa, which is well over normal (16 kPa), elevated (17.3 kPa), and hypertensive crisis (24 kPa) systolic blood pressures in humans. Finally, an in vivo swine model was used to assess the feasibility of using the newly developed two‐glue system for an endovascular anastomosis. X‐ray imaging confirmed that the anastomosis was made successfully without postoperative bleeding complications and the procedure was well tolerated. In the future, more studies are required to evaluate the performance of the developed sealants under various temperature and humidity ranges.

**Abbreviations**
%MA: percent of methacryloyl functionalization
ASTM: American Society for Testing and Materials
DPBS: Dulbecco's phosphate‐buffered saline
GelMA: gelatin methacryloyl
HUVEC: human umbilical cord endothelial cell
mELP: methacryloyl elastin‐like polypeptide

## 1 INTRODUCTION

When blood flow fails to satisfy metabolic demand, resultant ischemia leads to eventual cell death. The most frequent clinical scenario demonstrating this pathophysiology is arterial stenosis upstream of the tissue capillary bed due to atherosclerosis (lipid deposition into the vessel lumen). Any organ supplied by such an arteriovenous circuit is susceptible, as evidenced by common ailments like stroke, myocardial infarction, and distal extremity gangrene. Current revascularization procedures, including intraluminal stents and artery‐to‐artery bypass, have shown some benefits, but are not without limitations.^1^, ^2^, ^3^ Cerebral ischemia from intracranial atherosclerosis, in particular, remains difficult to treat. Stenting a diseased cerebral artery risks apposing plaque against small perforating branches and occluding them. In addition, intracranial bypass is technically challenging and carries high preoperative morbidity, in part due to infarcts occurring while the recipient vessel is clamped and the anastomosis sutured in. To overcome these limitations, one treatment option is to use a bypass approach whereby clamp time can be eliminated.

Identifying the optimal medical device to secure successful anastomosis requires a detailed evaluation of the required characteristics. Wound closure devices are utilized as physical barriers in order to decrease the tension on wound edges and to hold them together for a better sealing.^4^, ^5^ Proper sealing is essential to prevent postoperative complications such as infection and dehiscence.^6^, ^7^ The ideal wound closure devices should maintain their structural integrity, mechanical strength, and tissue adhesion under applied stress and/or changes in temperature and humidity.^8^, ^9^ In addition, biocompatibility and biodegradability are additional important characteristics of wound closure devices.^10^ Sutures, staples, strips, and hydrogel‐based sealants are examples of medical devices that have been used so far for wound closure with distinct advantages and disadvantages.^5^, ^11^

Despite the emergence and rapid growth of hydrogel‐based surgical sealants since early 2000s, sutures and staples are projected to continue dominating the wound closure market for the upcoming decade. This is largely due to their relatively lower cost and ease of use as compared to the hydrogel‐based sealants. However, sutures and staples are not suitable for all types of tissues or applications. For example, they can cause tissue damage upon insertion, leading to infection and further complications. Furthermore, internal organs such as the lungs, bladder, and blood vessels undergo changes in volume and pressure throughout their normal functions. Sutures and staples are not compatible with these organs as they may limit the natural tissue movement and function, and cause stress (damage) to the tissues.^6^, ^12^ To overcome these limitations, several formulations of sealants have been developed, specifically for vascular applications, such as poly(glycerol sebacate) acrylate based sealant, SETALIUM, and bovine serum albumin‐glutaraldehyde‐based sealant, BioGlue. These sealants have shown promising results in carotid artery and jugular vein defects in a porcine model without thrombus formation or stenosis.^13^, ^14^ In addition, there are a number of fibrin‐based sealants used to strengthen vascular suture lines.^15^ Although these sealants are designed to promote coagulation while maintaining biocompatibility and biodegradability, they suffer from low stiffness and elasticity and limited adhesion to the wet biological surfaces.

To address the above‐mentioned limitations, in this study, we developed a new approach for endovascular anastomosis based on using two types of surgical sealants with high tissue adherence and specific mechanical properties to tolerate high pressure and/or stress. The first glue was composed of gelatin methacryloyl (GelMA), a functionalized derivative of collagen, and the second sealant was made of GelMA and methacryloyl elastin‐like polypeptide (mELP), a 365‐amino acid (aa) recombinant elastomer designed to mimic the properties of natural elastin. In our previous work, we formed highly elastic ELP hydrogels through disulfide bond formation upon exposure to UV light.^16^ In this study, we functionalized the lysine, serine, and tyrosine residues on ELP with methacrylamide and methacrylate groups, respectively, to obtain mELP, an elastomer capable of forming a stable and elastic photocrosslinkable hydrogel standalone upon exposure to LED light at 450–500 nm wavelength. However, pure mELP‐based hydrogels had insufficient mechanical characteristics to serve as a surgical sealant. Therefore, we introduced a photocrosslinkable secondary hydrogel (GelMA) in order to provide the necessary stiffness for endovascular anastomosis. The mechanical properties of the engineered photocrosslinkable composite glue were optimized to mimic the native vascular tissue. In addition, its adhesive properties were optimized to obtain high adhesion to vascular tissue while retaining biocompatibility in vitro. The mechanical properties and adhesion of the sealants were evaluated through experimental protocols established by the American Society for Testing and Materials (ASTM). Finally, an ex vivo experiment and two in vivo tests using rat and swine models were performed to evaluate the vascular retention and adhesion of the developed system for the proposed application of anastomosis in cerebrovascular ischemia.

## 2 RESULTS AND DISCUSSION

### 2.1 Synthesis and structural characterization of GelMA/mELP hydrogels

**FIGURE 1:**
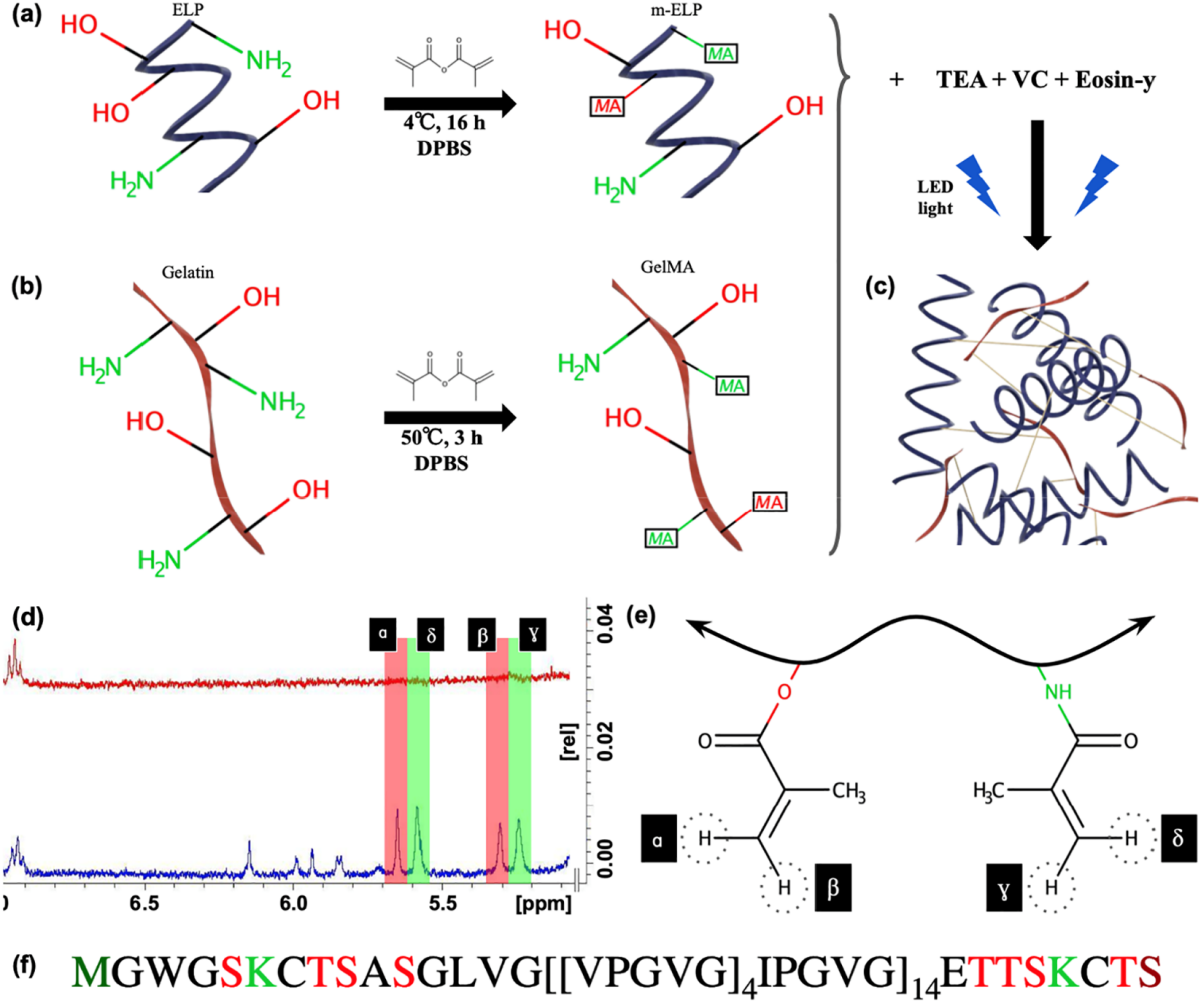
Molecular characterization of methacryloyl elastin‐like polypeptide (mELP) and gelatin methacryloyl (GelMA) biopolymers. (a) Methacryloyl functionalization reaction of elastin‐like polypeptide (ELP) to yield mELP with 40% methacrylamide functionalization of primary amine groups of lysine and N‐terminal methionine residues and 8% methacrylation of hydroxyl groups of the serine and threonine residues. (b) Methacryloyl functionalization of gelatin to produce GelMA with 82% methacrylamide functionalization. (c) The modified polymers were dissolved in a photoinitiator solution containing triethanolamine (TEA), N‐vinylcaprolactam (VC), and Eosin Y and photocrosslinked with a visible light to form a solid and adhesive sealant. (d) Proton nuclear magnetic resonance (^1^H‐NMR) spectra of ELP (red) and mELP (blue), confirming the modification of the polymer. (e) Each of the two characteristic protons of both methacrylate and methacrylamide groups are labeled as shown on the ^1^H‐NMR data. (f) The ELP sequence. The lysine (light green) and the N‐terminal methionine (dark green) residues contain primary amine (—NH_2_) groups that allow methacrylamide functionalization. The serine and threonine residues (red) have hydroxyl (—OH) groups that allow methacrylate functionalization. The C‐terminal serine (dark red) has two potential methacrylation sites

In this study, we developed new formulations of elastic sealants to be used in an endovascular anastomosis procedure for the treatment of cerebrovascular ischemia. These hydrogel‐based sealants were engineered using two modified biopolymers: mELP (Figure 1(a)) and GelMA (Figure 1(b)). mELP is a photocrosslinkable, recombinant elastomer produced by genetically modified *Escherichia coli*; it serves as an elastic peptide that provides penetrability and extensibility.^16^ On the other hand, GelMA is a photocrosslinkable biopolymer comprised of modified gelatin and it provides physiological cell binding motifs and protease‐sensitive degradation sites as well as high mechanical strength and adhesion.^17^ Here, we incorporated both mELP and GelMA into a polymeric network, enabling the modulation of several features such as degradation rate, mechanical properties, and tissue adhesion of the resulting composite glues. In addition, we used a visible light activated photoinitiator system to minimize the biosafety concerns associated with UV light such as DNA damages.^18^ In particular, we utilized the Type 2 initiator Eosin Y, the co‐initiator triethanolamine (TEA), and the co‐monomer N‐vinylcaprolactam (VC) for the photocrosslinking (Figure 1(c)). Briefly, visible light excites dye molecules of Eosin Y into a triplet state, which abstracts hydrogen atoms from TEOA. The deprotonated radicals initiate vinyl‐bond crosslinking with VC via chain polymerization reactions, which leads to accelerated gelation.^19^

To verify the degree of methacryloyl substitution of each biopolymer, proton nuclear magnetic resonance (^1^H‐NMR, 400 MHz) analysis was used. Results showed the emergence of the methacrylate (ɑ/β) and the methacrylamide (γ/δ) proton peaks for mELP (Figure 1(d,e)) within 5.2–5.7 ppm range. Knowing the stoichiometric amounts of lysine, methionine, serine, and threonine residues, we used a reference molecule (PEG_2000_) to determine the percentages of modified amino acids. ^1^H‐NMR analysis of mELP showed 40% degree of methacryloyl functionalization of lysine and terminal methionine amines to form methacrylamide groups and 8% degree of methacryloyl functionalization of serine and threonine residues to form methacrylate groups. These values are in agreement with the degree of methacryloyl functionalization of tropoelastin to yield methacrylated tropoelastin (MeTro) with 44% methacrylation, following the same synthesis method.^20^ The engineered ELP sequence is shown in Figure 1(f). Similarly, the degree of methacrylamide functionalization for GelMA was quantified to be 82%, which is in agreement with the previously published results following similar synthesis protocol.^17^

### 2.2 Mechanical characterization of the engineered hydrogel‐based sealants

**FIGURE 2:**
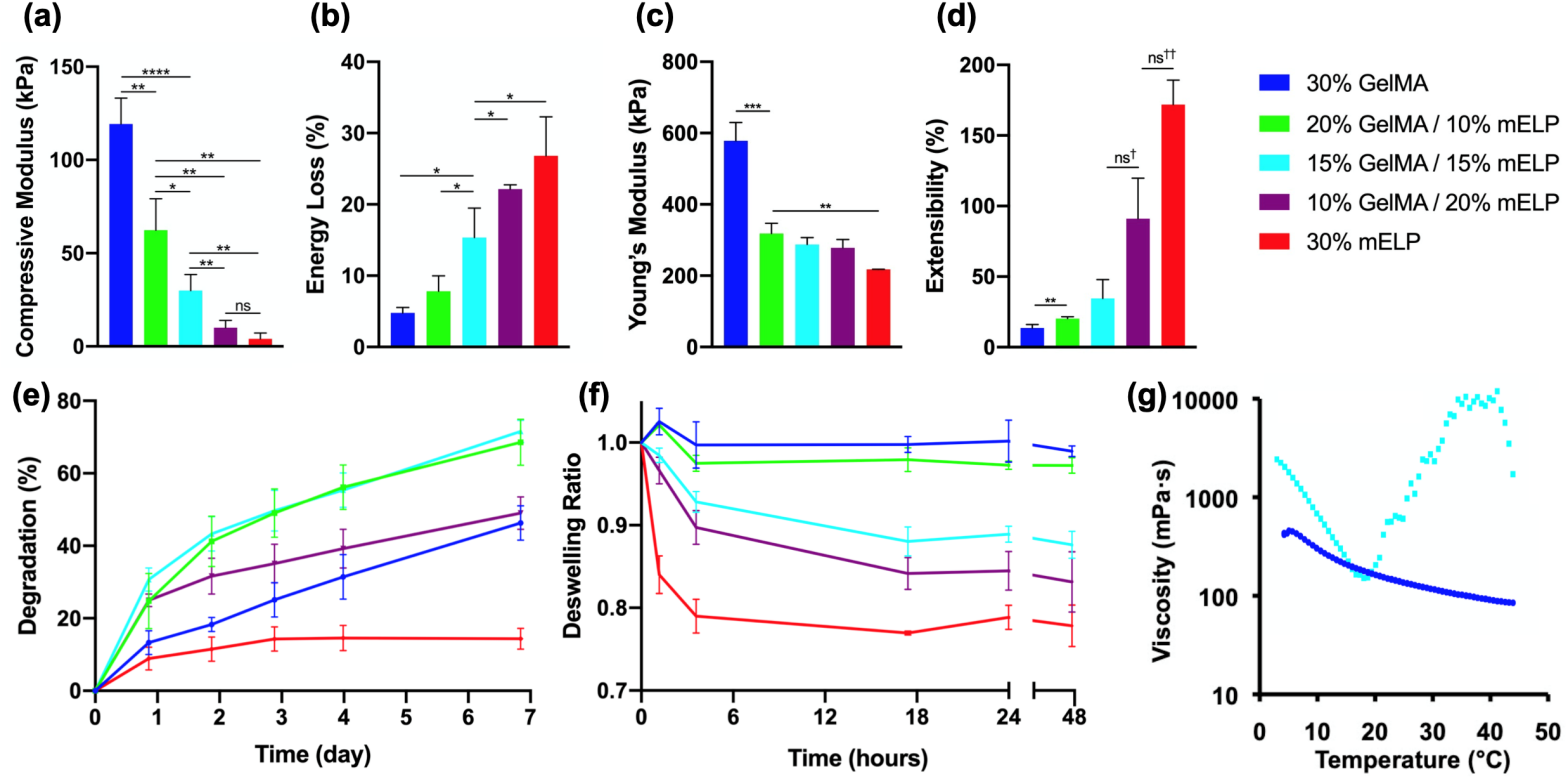
Physical characterization of the engineered hydrogel‐based sealants. (a) Compressive modulus and (b) energy loss percentage of the hydrogel formulations made of various concentrations of methacryloyl elastin‐like polypeptide (mELP) and gelatin methacryloyl (GelMA) obtained from the unconfined cyclic compression test. (c) Young's modulus and (d) maximal extensibility obtained from the tensile test (ns^†^ *p* = .0887, ns^††^ *p* = .0528). (e) In vitro degradation profiles of the hydrogel samples in 20 μg/ml collagenase solution in Dulbecco's phosphate‐buffered saline (DPBS) solution at 37°C. (f) Deswelling behavior of the hydrogel samples in DPBS at 37°C. (g) Temperature‐dependent viscosity profiles of 15% GelMA/15% mELP composite prepolymer solution (light blue) and pure 30% GelMA prepolymer solution (dark blue). (*p < .05, **p < .01, ***p < .001, and ****p < .0001)

Mechanical properties of the hydrogel‐based sealants were characterized through tensile and cyclic compression tests. As shown in Figure 2(a), the unconfined compressive moduli of the 15% GelMA/15% mELP composite sealant (30 ± 9 kPa) was between the pure mELP hydrogel (4 ± 3 kPa) and the pristine GelMA hydrogel (119 ± 14 kPa). Increasing GelMA concentration significantly improved both the stiffness (Figure 2(a)) and toughness (Supp. Figure 4) of the resulting composite hydrogels. Regarding the cyclic compression test, the pure GelMA sample presented the smallest energy loss of 4.8 ± 0.8% after 10 cycles of loading/unloading while 15% GelMA/15% mELP composite showed 15.4 ± 4.1% energy loss (Figure 2(b)).

Tensile tests on the hydrogels revealed that the Young's modulus (Figure 2(c)) and the extensibility (Figure 2(d)) could be tuned by varying the GelMA/mELP ratio at a constant total polymer concentration of 30% (w/v). Representative stress–strain curves for all the formulations are provided in Supp. Figure 5. The Young's modulus of pure GelMA and pure mELP hydrogels were 581 ± 51 and 218 ± 1 kPa, respectively. Although the Young's modulus of the engineered composite hydrogels decreased with increasing the mELP concentrations, their extensibility exhibited an opposite trend, which was later determined to be a critical factor for the anastomosis application. The pure GelMA hydrogel could be extended up to 13.6 ± 2.5% before rupture while the pure mELP hydrogel had an extensibility of 172 ± 17%. Prior studies also demonstrated 163 ± 11% extensibility for hydrogels containing 15% (w/v) ELP.^21^

It was notable that four of the five prepolymer solutions could be easily handle; however, the pure mELP prepolymer solution was too viscous to be pipetted/injected and thus was considered impractical for clinical settings. Yet, incorporation of mELP biopolymer in the composite hydrogel significantly improved the elasticity (i.e., extensibility) after exposure to visible light and crosslinking.

### 2.3 In vitro swelling and degradation of the engineered hydrogel‐based sealants

Another benefit for the use of hydrogels as surgical sealants is controlled degradation in wet environments. Therefore, we aimed to investigate the in vitro degradation profiles of the engineered hydrogels in collagenase Type II solution. Results demonstrated that the in vitro degradation was consistently higher for the composite hydrogels compared to that of pure GelMA or mELP alone which corresponded to 46 ± 5% and 14 ± 3%, respectively, after 7 days of incubation in the collagenase Type II solution (Figure 2(e)). The 15% GelMA/15% mELP composite had the highest 7‐day degradation at 72 ± 3%. The tunable degradation rate of the engineered sealants, based on GelMA and mELP concentrations, make them suitable for a wide range of surgical applications.

We also determined the swelling ratios of the resulting hydrogels at various time points, throughout their incubation in Dulbecco's phosphate‐buffered saline (DPBS) at 37°C. In general, the results for all samples showed initial decrease in swelling (i.e., deswelling) (Figure 2(f)). This was the expected behavior for both types of biopolymers. Previous works have shown that the swelling decreased with an increase in total concentration of GelMA, and the swelling ratio approached zero at 20% total polymer concentration.^22^ Our results demonstrated that hydrogels formed by using pure GelMA had minimal deswelling compared to the hydrogel compositions containing higher mELP concentration, while the 15% GelMA/15% mELP formulation had a deswelling ratio of 0.88 ± 0.02 at 24 h and remained stable afterward. Control over the swelling ratio of the composite hydrogels is advantageous for their use in medical field since it can be fine‐tuned based on their final application. Rheology analysis also showed that the optimal working temperature for the 15% GelMA/15% mELP solution was between 15 and 20°C due to the cold soluble nature of mELP in an aqueous solution (Figure 2(g)).

### 2.4 In vitro and ex vivo adhesive properties of the engineered hydrogel‐based sealants

In general, high tissue adhesion can prevent biomaterial detachment from target tissues in vivo and ultimately promote wound closure and biointegration. Herein, we examined several critical properties for effective tissue sealing including burst pressure and adhesion strength in accordance with the ASTM standards for biological adhesives. The in vitro adhesion strength and sealing properties of GelMA/mELP composite hydrogels at various concentrations of GelMA and mELP were investigated.

**FIGURE 3:**
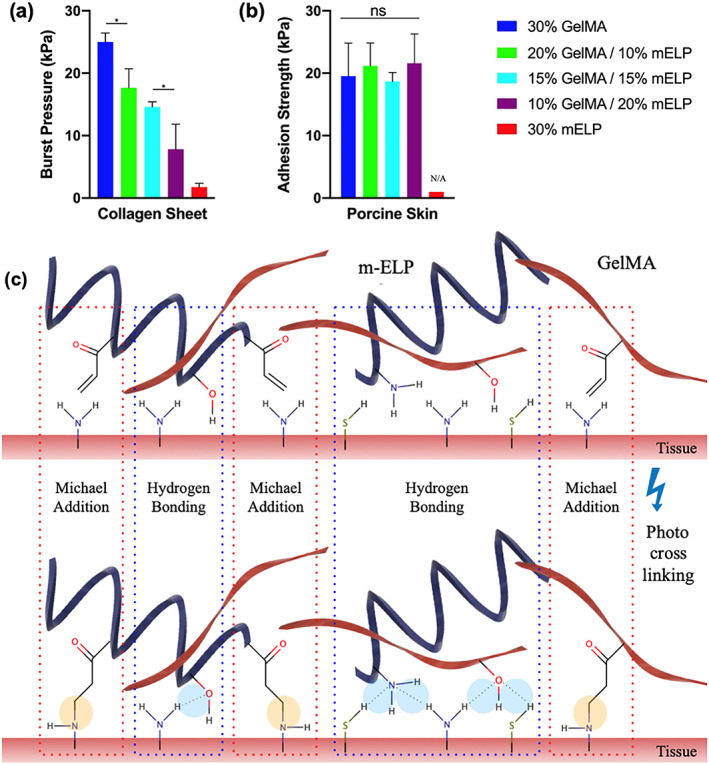
Adhesion strength of the hydrogel formulations and mechanism of interactions. (a) The in vitro burst pressure values obtained based on standard burst pressure tests on sealants formed by using various concentrations of methacryloyl elastin‐like polypeptide (mELP) and gelatin methacryloyl (GelMA). (b) Adhesion strength of the hydrogels on porcine skin obtained by using a standard wound closure. The pure mELP gel was not tested as it lacked the necessary injectability for the precise application of the prepolymer solution for the wound closure test. (c) Schematic of interactions between mELP and GelMA polymer chains and tissue before (top) and after (bottom) crosslinking. Two most prominent interactions are covalent linkages and hydrogen bonding. The covalent linkages are between the methacryloyl alkenes on the polymers and the primary amines on the tissue surface, and form through Michael addition reactions. (*p < .05)

The results showed that the burst pressure significantly increased from 1.8 ± 0.6 kPa (~13 mmHg) for the pure 30% mELP hydrogels to 25 ± 1 kPa (~187 mmHg) for the pure 30% GelMA hydrogel (Figure 3(a)), which is above human systolic blood pressure during hypertensive crisis (180 mmHg). In addition, no significant difference in adhesive properties of different composite hydrogels was observed, via wound closure test using porcine skin as the biological substrate (Figure 3(b)). The interactions between the polymer chains and tissue before and after photocrosslinking are shown in Figure 3(c), demonstrating the formation of different covalent linkages and hydrogen bonding between the tissue and hydrogel upon photopolymerization.

**FIGURE 4:**
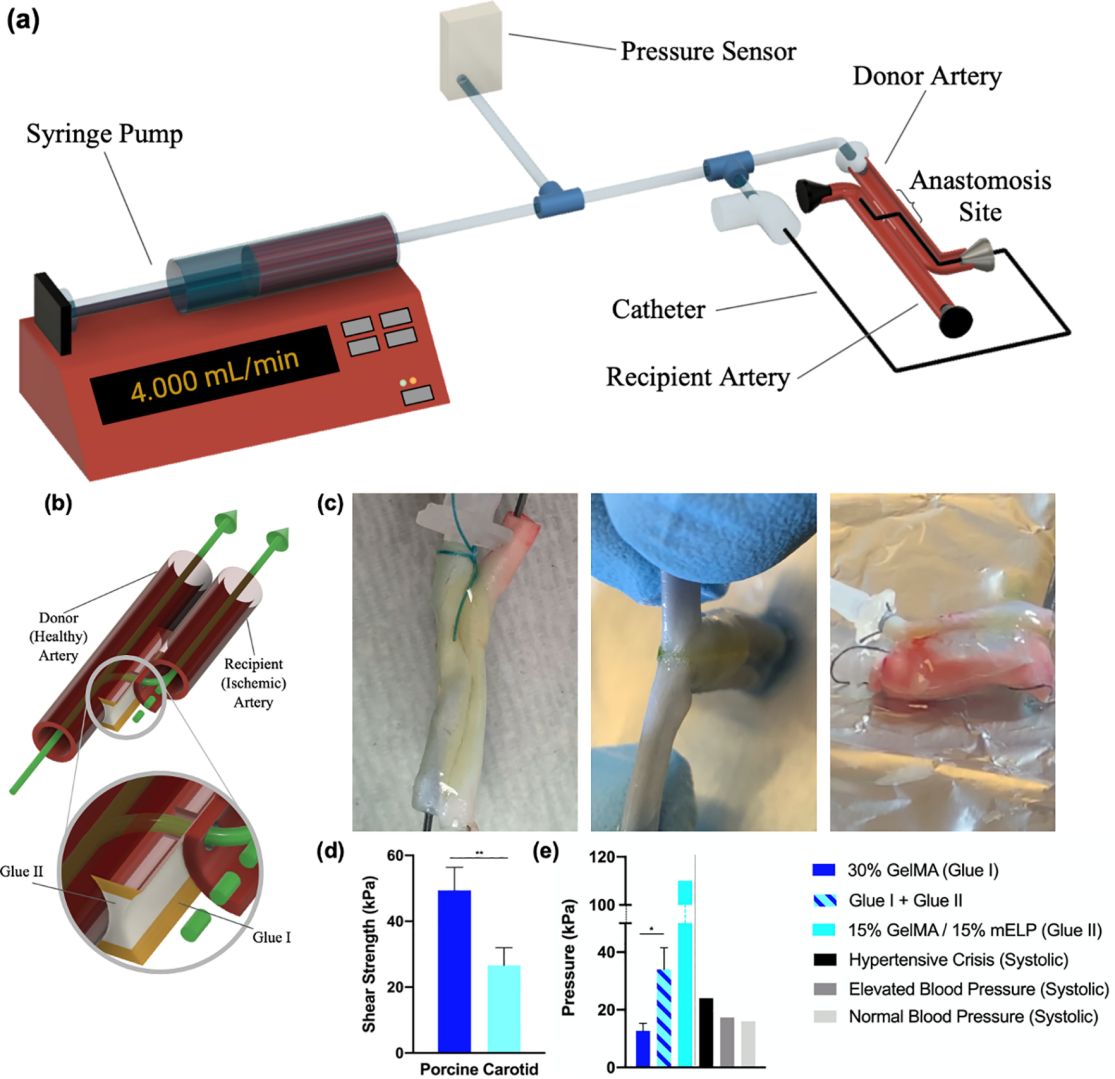
Ex vivo endovascular anastomosis model and shear test. (a) Experimental setup. (b) 3D schematic of the proposed two glue‐based system. The elastic composite formulation is applied between the arteries to allow penetration through the gel. The stiff pure gelatin methacryloyl (GelMA)‐based formulation is then applied around to stabilize and fortify the anastomosis site. (c) Two carotid segments were glued together; the anastomosis procedure was complete, and a catheter was inserted (left). The glue sealed the area surrounding the anastomosis and prevented fluid leakage; the arteries could not be pulled apart with ease (center). Using this ex vivo anastomosis model, both arteries expanded up to four to six times in diameter before the glue system failed and burst (right). (d) Shear strength of the applied glues on porcine carotid arteries. (e) The maximal intra‐arterial pressures achieved with the ex vivo endovascular anastomosis model, in comparison to human systolic blood pressures at various stages (*p < .05, **p < .01)

Next, an ex vivo anastomosis model was developed to further evaluate the adhesive strength and sealing functionality of the hydrogels (Figure 4(a,b)). The results showed that the pure GelMA bioadhesives failed to resist stress at an intra‐arterial pressure of 12.7 ± 2.6 kPa. We hypothesized that this was due to the previously determined low extensibility (brittleness) of the GelMA hydrogel; the anastomosis resulted in cracks within the sealant that led to leakage. Next, we applied the 15% GelMA/15% mELP composite hydrogel, which showed optimal adhesion and elasticity with a burst pressure value of 110 kPa (~825 mmHg). The rationale behind choosing this formulation is due to its mechanics (higher elasticity relative to 20% GelMA/10% mELP, Figure 2(a–d)) and adhesion (higher adhesion relative to 10% GelMA/20% mELP, Figure 3(a)). However, the fast rate of in vitro degradation of the 15% GelMA/15% mELP composite bioadhesive, in conjunction with the slow degradation rates of the pure GelMA adhesive brought us the idea of the circumferential application of the pure GelMA formulation. Therefore, we introduced a two glue‐based system in which we first applied 15% GelMA/15% mELP composite bioadhesive (i.e., Glue II) between two vessels to allow for penetration, followed by the circumferential application of the stronger and highly adhesive pure GelMA hydrogel (Glue I) for further fortification and sealing (Figure 4(b,c)). The application of two hydrogel‐based glues on the same endovascular anastomosis model achieved an intra‐arterial pressure of 34.0 ± 7.5 kPa, which is well over normal, elevated, and hypertensive crisis systolic blood pressures in humans at 16, 17.3, and 24 kPa, respectively (Figure 4(e)). In addition, the lap shear strengths of the pure 30% GelMA (Glue I) and the composite 15% GelMA/15% mELP bioadhesives (Glue II) were calculated. Shear adhesive strength of glue II was significantly lower (26.7 ± 5.4 kPa) than that of glue I (49.4 ± 7.0 kPa) (Figure 4(d)).

### 2.5 In vitro cytocompatibility of the engineered hydrogel‐based sealants

**FIGURE 5:**
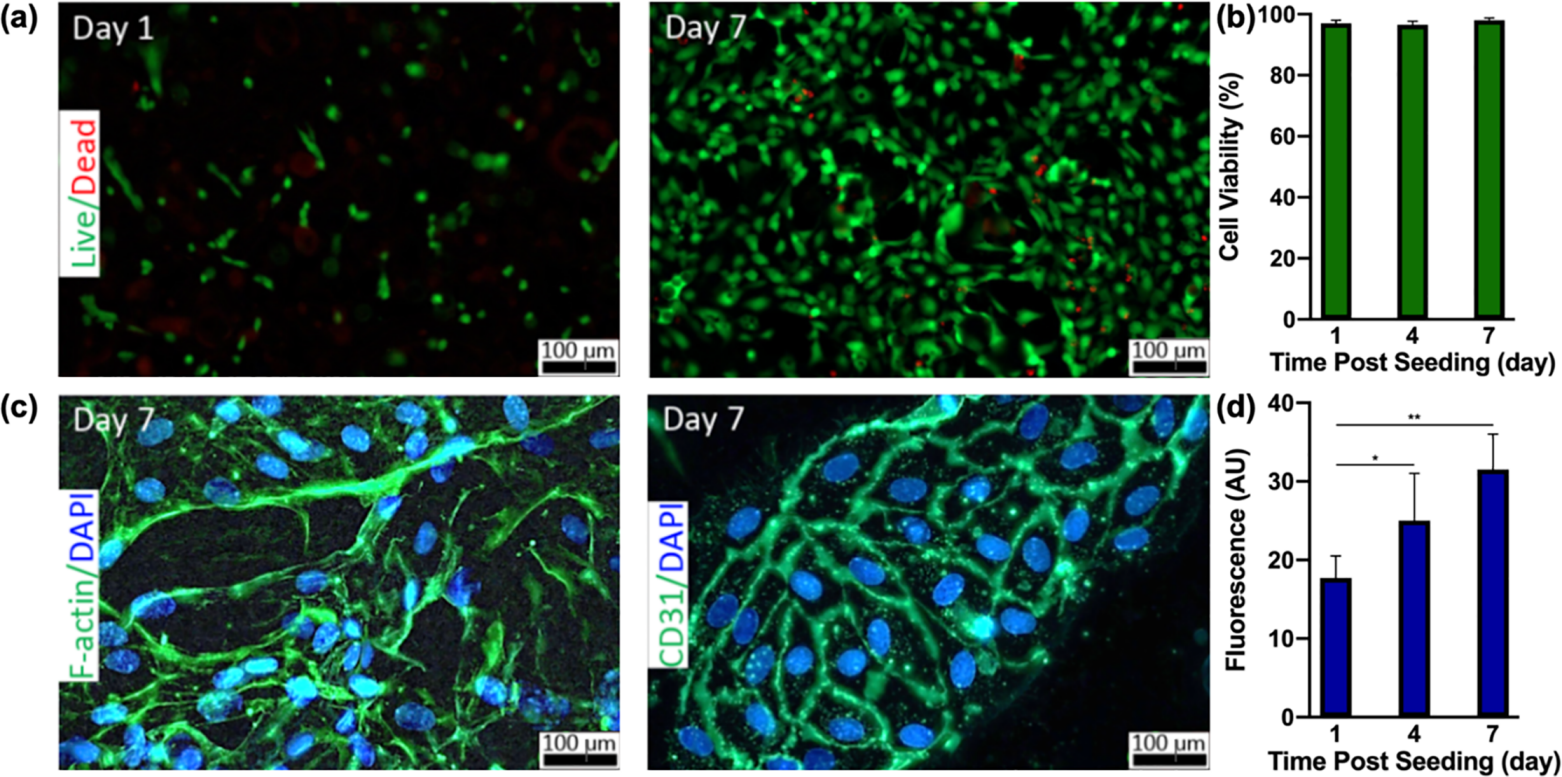
In vitro biocompatibility of the engineered sealant seeded with human umbilical vein endothelial cells (HUVECs). (a) Representative live/dead images from the cells seeded on hydrogels on Days 1 and 7 post‐seeding. (b) Quantification of cell viability based on the live/dead images on Days 1, 4, and 7 days after seeding. (c) Representative images based on F‐actin/DAPI (left) and CD31/DAPI (right) staining on Day 7 post‐seeding. (d) The results of PrestoBlue assay on cell‐seeded hydrogel on Days 1, 4, and 7, showing a significant increase in cellular metabolic activity and proliferation over time (*p < .05, **p < .01)

The optimal bioadhesive for anastomosis should be cytocompatible. It should also permit cell proliferation within the injured tissue for faster integration and healing. Therefore, we aimed to evaluate the in vitro cytocompatibility of the engineered 15% GelMA/15% mELP composite bioadhesives (Glue II) via live/dead and PrestoBlue assays, as well as actin/DAPI and CD31/DAPI staining of human umbilical vein endothelial cells (HUVECs) seeded on the surface of the bioadhesives. Cytotoxicity of hydrogels at various concentrations of GelMA has previously been assessed by our group using various cell types, and GelMA hydrogel has been shown to be cytocompatible.^23^, ^24^ ELPs have been of particular interest in recent years due to their unique mechanical properties. However, cell viability has been shown to drop down to as low as 80% in extracellular scaffolds containing ELP. In literature, several modifications such as incorporation of fibronectin has been suggested to improve cytocompatibility.^25^, ^26^ The results of the in vitro cytocompatibility tests for 15% GelMA/15% mELP demonstrated desirable proliferation and spreading of the surface seeded and metabolically active HUVECs. The cell viability remained very high (>97%) throughout the 7 days of culture (Figure 5(a,b)). The cells were also adhered and spread on the bioadhesive over 7 days of culture (Figure 5(c)). In addition, the metabolic activity (fluorescence arbitrary units [a.u.]) of the cells increased significantly from Day 1 (17.7 ± 2.8 a.u.) to Day 4 (25.0 ± 6.0 a.u.), and to Day 7 (31.5 ± 4.5 a.u.) (Figure 5(d)). These results together demonstrated the in vitro cytocompatibility of the engineered composite hydrogels.

### 2.6 In vivo biocompatibility and biodegradation of the engineered hydrogel‐based sealants

**FIGURE 6:**
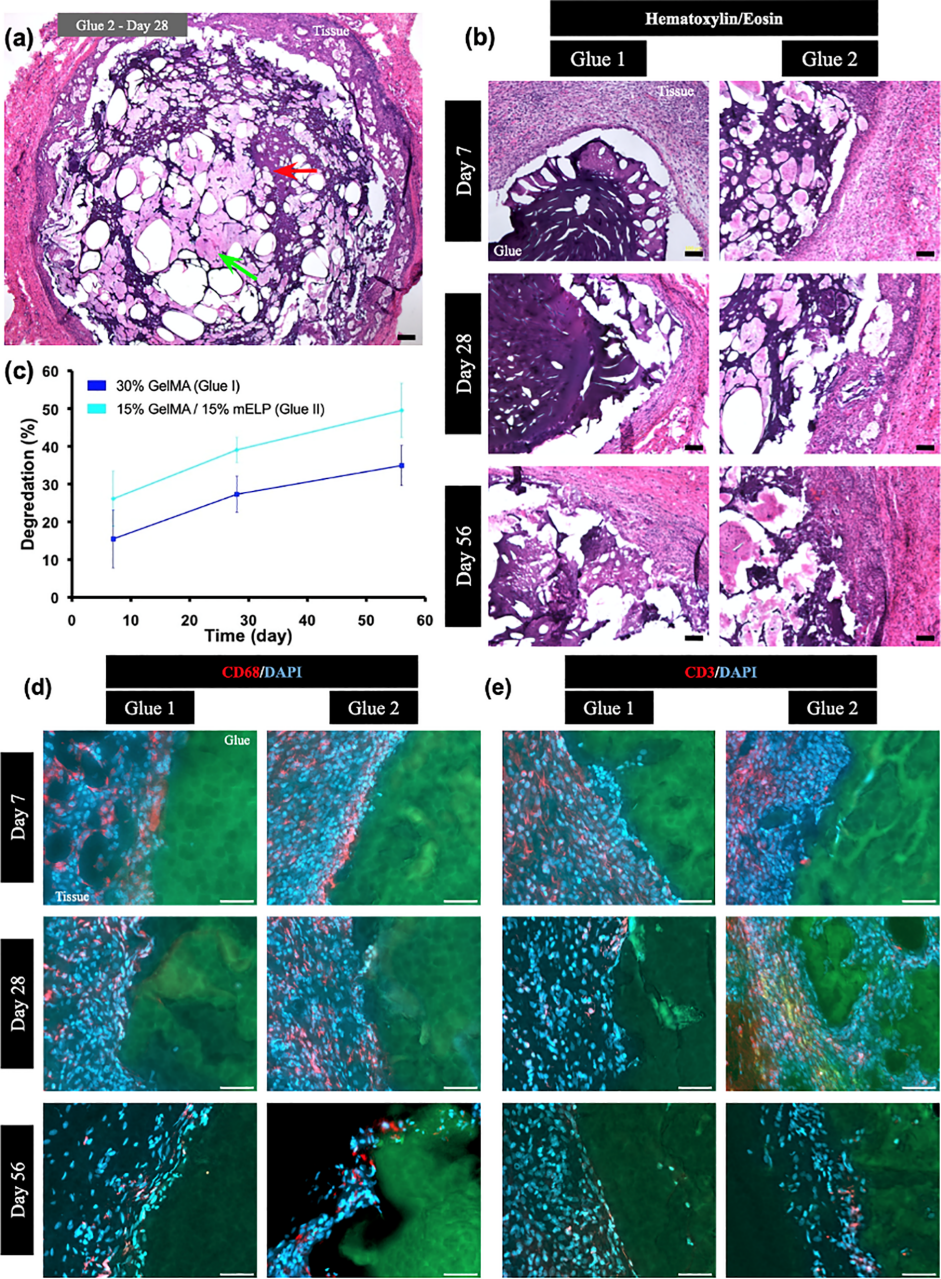
In vivo degradation and biocompatibility of the engineered sealant using a rat subcutaneous implantation model. (a) A representative hematoxylin/eosin‐stained image from a 15% gelatin methacryloyl (GelMA)/15% methacryloyl elastin‐like polypeptide (mELP) hydrogel (Glue II) explanted on Day 28. Degrading hydrogel is shown with red arrow and tissue infiltrated inside the hydrogel is shown with green arrow (scale bar: 500 μm). (b) Representative hematoxylin/eosin‐stained images from the glue/tissue interfaces at different explantation times including Days 7, 28, and 56. For both formulations, the hydrogels degraded by Day 56 (scale bars: 100 μm). (c) The in vivo degradation profiles for both formulations, showing faster degradation for the 30% pure GelMA hydrogel as compared to the composite hydrogel. (d,e) Immunohistofluorescent analysis of subcutaneously implanted hydrogels for local lymphocytes (CD3) and macrophage infiltration (CD68) at Days 7, 28, and 56, indicating initial inflammatory response for both formulations that diminished over time (scale bars: 50 μm). Green, red, and blue colors represent the autofluorescent hydrogels, the immune cells, and cell nuclei (DAPI), respectively

The in vivo degradation and biocompatibility of the engineered bioadhesives were studied using a rat subcutaneous implantation model. Hematoxylin and eosin staining of the explanted samples revealed that tissue/cell ingrowth was observed inside the 15% GelMA/15% mELP hydrogels (Glue II) as early as Day 7 and increase over time as the hydrogel went through degradation (Figure 6(a,b)). However, the pure GelMA hydrogels (Glue I) maintained their overall structural integrity with limited cellular/tissue penetration up to Day 28 (Figure 6(b)). In general, both hydrogels showed consistent degradation over the course of the experiment, up to 56 days with a higher degradation rate for the composite hydrogel compared to pure GelMA hydrogel (Figure 6(c)). These results are consistent with the in vitro degradation results presented in Figure 2(e). Cryosectioned samples were also analyzed through immunohistofluorescent staining of macrophage and lymphocyte antigens, CD68 and CD3, respectively (Figure 6(d,e)). The fluorescence images revealed inflammation around the hydrogel implants, particularly for the 15% GelMA/15% mELP composite on Day 7 post‐surgery. However, the initial inflammatory response diminished by Day 28 (Figure 6(d,e)).

### 2.7 In vivo feasibility of the two hydrogel‐based glues using a nonsurvival anastomosis pig model

**FIGURE 7:**
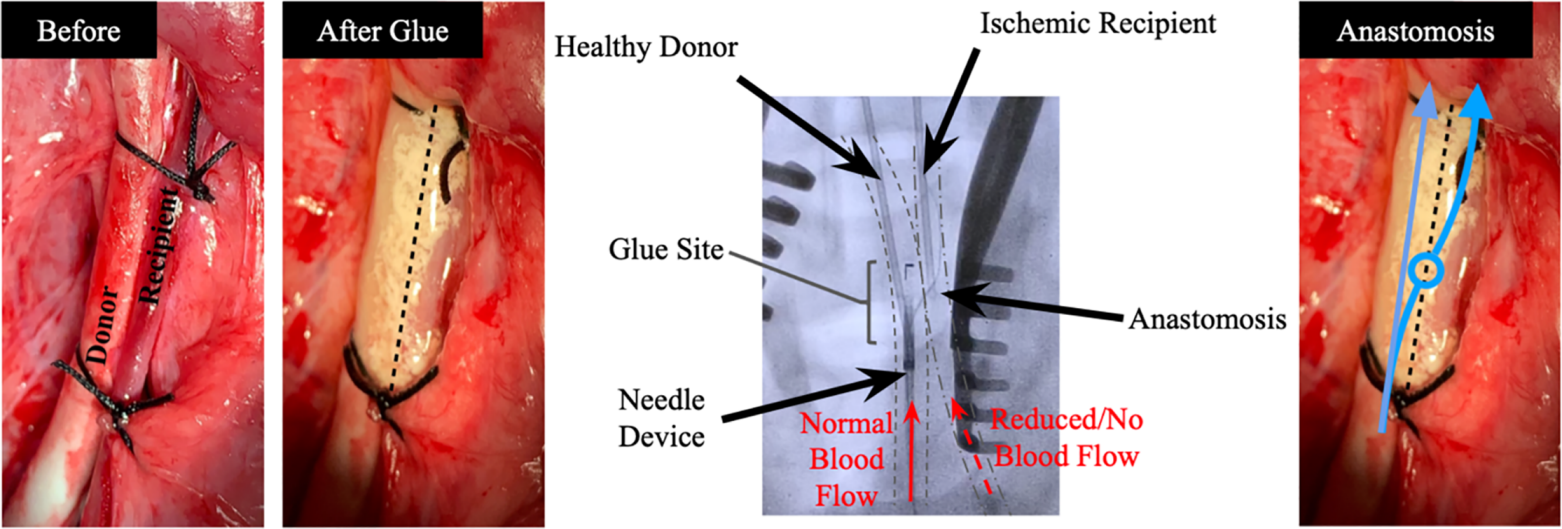
In vivo feasibility of the two hydrogel‐based glues using a nonsurvival anastomosis pig model. The arteries were tied together with sutures and glued in accordance with the described model for the two hydrogel‐based glues. The needle device was inserted into the bloodstream from the femoral artery and advanced toward the anastomosis site within the donor carotid artery. A successful anastomosis was performed through the composite glue into the recipient artery

A newly developed in vivo swine model was used to assess the feasibility of the two hydrogel‐based glues for the endovascular anastomosis procedure. Two arteries were surgically exposed and placed closely in parallel. The two hydrogel‐based glues were then applied at the anastomosis site under homeostatic conditions and crosslinked via exposure to visible light. The anastomosis was successfully performed without bleeding complications and confirmed via x‐ray imaging (Figure 7). The procedure was well tolerated. However, it is notable to mention that the malfunction of the Outback needle device in one incident was emphasized the need for a novel, purpose‐built instrument to create an anastomosis using the intraluminal, transmural approach. Future studies will explore radio frequency ablation for this specific purpose, including how the hydrogel responds to temperature and humidity changes.

## 3 METHODS

### 3.1 Synthesis of GelMA

GelMA was synthesized as explained previously.^17^ Briefly, gelatin from cold water fish skin was dissolved in DPBS (10% w/v). Then, methacrylic anhydride (Sigma‐Aldrich) was added dropwise (8% v/v) at 60°C and the mixture was allowed to react for 3 h under continuous stirring. The reaction was then stopped by 1:4 dilution with DPBS. Finally, the solution was dialyzed against deionized water for 7 days, frozen at −80°C for 2 h, and desiccated for 5 days to yield high GelMA.

### 3.2 Synthesis of mELP

Plasmid inserted, kanamycin resistant *E*. *coli* strain genetically modified to encode elastin‐like polypeptide (ELP) was removed from −80°C storage and inoculated in 10 ml Luria‐Bertani broth. The starter culture was left overnight on a shaker incubator at 37°C, 190 rpm. The starter culture was then transferred into 1.5 L Terrific Broth containing kanamycin (50 mg/L) and placed back in the shaker incubator for 24 h. The liquid culture was then centrifuged at room temperature at 17,000*g* for 20 min. The pellet was collected, placed in lysis buffer (5.84 g‐NaCl/L, 0.48 g‐MgCl_2_/L, 1.00 ml‐βME/L in [1x] TE buffer) at 4°C, and kept in the refrigerator overnight. The mixture was then sonicated and refrigerated overnight. Inverse transition cycling was applied with one cycle of cold and warm spin per day for 4 days. After the fifth cold spin, the solution was pipetted into dialysis membranes and dialyzed against milli‐Q water (changed twice per day) at 4°C for 4 days.^16^, ^21^ The purified solution was frozen at −80°C and lyophilized to yield ELP.

Purified ELP was then dissolved in DPBS (10% w/v) at 4°C and methacrylic anhydride was added dropwise to a 15% v/v final concentration. The mixture was continuously stirred in an ice bath and was allowed to react for 16 h. The mixture was then diluted into 4x volume with cold DPBS and dialyzed in a dialysis cassette against milli‐Q water (changed twice per day) at 4°C for 4 days. The purified solution was frozen at −80°C and lyophilized to yield mELP.

### 3.3 ^1^H‐NMR characterization of GelMA and mELP polymers

There are well‐established methods to determine the degree of methacryloyl functionalization (%MA) of the extensively studied polymers such as GelMA, which involves the quantification of the diminishing free lysine in ^1^H‐NMR spectra with increasing degree of methacryloyl substitution.^27^, ^28^ Here, we developed a new strategy to identify the molecular characteristics of mELP because of the (i) low lysine content of ELP (2 units per chain) and (ii) high hydroxyl (i.e., serine and tyrosine) to amine (i.e., lysine) residue ratio throughout the peptide sequence. Similar to other studies on polymers, we used polyethylene glycol 2000 (PEG_2000_, Sigma, CAS: 25322‐68‐3) as the reference molecule (standard) and D_2_O as the solvent for calculation the degree of methacryloyl substitution (i.e., methacrylamide functionalization of lysine and methacrylate functionalization of serine and tyrosine) based on the following equation.

$$\%\;\text{Methacryloyl Functionalization}=\frac{\int \left(H*+H*\right)}{\int \left(\mathrm{PEG}\right)}\cdot \frac{1}{\psi }\cdot \frac{179H_{\mathrm{PEG}}}{2H{*}_{m-\mathrm{ELP}}}$$

Here, the H* and H*′ are the two terminal alkene protons of a methacryloyl group that present as two distinct singlets of exactly the same intensity at slightly different chemical shifts due to stereochemistry around the alkene. The terms *n*_*PEG*_ and *n*_*m‐ELP*_ are the controlled number of moles of PEG_2000_ and mELP, respectively, in the spectra. Ψ is the number of relevant residues per ELP chain; for methacrylamide functionalization, Ψ = 2 (2 lysine residues in sequence) and for methacrylate functionalization, Ψ = 9 (5 serine and 4 tyrosine residues in sequence). PEG_2000_ integral at 3.47 ppm was nominally integrated for 179 aliphatic protons to obtain the total methacryloyl group content (i.e., aforementioned H* and H*′ peaks). For GelMA, the same calculation was carried out using residue per unit mass (i.e., 2.5 × 10^−4^ mol‐lysine/mg‐gelatin) instead of residue per chain.

### 3.4 Engineering the hydrogel‐based sealants

A photoinitiator stock solution based on TEA and VC was prepared by dissolving 3.75% w/v TEA and 2.5% w/v VC in DPBS. A second stock was contained 1 mM Eosin Y in DPBS. Both stocks were kept in dark at 4°C. TEA‐VC and Eosin Y solutions were then mixed at 4:1 ratio to obtain the photoinitiator solution. The bioadhesive precursor solutions were prepared by first cooling down the photoinitiator solution to 15°C, followed by the addition of mELP and subsequent vortexing (3000 rpm) at 15°C for 30 min until full dissolution. Next, GelMA was added and vortexed (3000 rpm) at 15°C until full dissolution. The precursor solutions were prepared by varying concentrations of GelMA and mELP polymers at a constant total polymer concentration of 30% w/v. The solution was then photocrosslinked with blue LED light (450–500 nm) for 160 s using an LS1000 Focal Seal Xenon Light Source (100 mW/cm^2^, Genzyme).

### 3.5 Mechanical characterization

The prepolymer solutions were pipetted into polydimethylsiloxane molds of rectangular geometry (12 × 4.5 × 1 mm) for tensile testing and of cylindrical geometry (*d*: 5 mm, *h*: 4 mm) for cyclic compression testing and crosslinked as explained above. The precise dimensions of the fabricated hydrogels were obtained using a digital caliper. The tensile and the unconfined cyclic compression tests were performed using an Instron 5542 mechanical tester. For tensile tests, rectangular hydrogel samples were placed between double‐sided polyethylene terephthalate based tapes and loaded to the mechanical tester. Samples were stretched longitudinally at a rate of 1 mm/min. Tensile strain (%) and tensile stress (kPa) were measured with a BlueHill Universal software. The extensibility percentage was determined by maximum strain and the Young's modulus was obtained by calculating the slope of stress–strain curves. For the unconfined cyclic compression test, the cylindrical hydrogel samples were placed between the compression plates of the mechanical tester. The samples were then compressed and decompressed for 10 cycles to a maximum strain of 40% at a rate of 5 mm/min. Compressive strain (%) and stress (kPa) were measured with a BlueHill Universal software. The unconfined compressive modulus was determined from the slope of the initial linear region of the stress–strain curve of the first cycle. The energy loss percentage was calculated by the area between the loading and unloading for each cycle.

### 3.6 In vitro swelling ratio and degradation test

For the swelling test, cylindrical samples were prepared as described previously, weighed (*w*_0_), and submerged in 1 ml DPBS in separate wells of a 24‐well plate. The samples were weighed after 2, 4, 16, 24, and 48 h with fresh DPBS added every interval. The deswelling ratio at each time point was defined as the ratio of the corresponding weight (*w*_*ti*_) to initial weight (*w*_0_).

$$\text{Deswelling}\;{\text{Ratio}}_{t-\mathrm{ti}}=\frac{w_{\mathrm{ti}}}{w_{0}}$$

For the in vitro degradation test, cylindrical samples were prepared as described previously, weighed (*w*_0_), and submerged in 1 ml DPBS solution containing 20 μg/ml collagenase Type II in separate wells of a 24‐well plate. The samples were weighed after 1, 2, 3, 5, and 7 days and at each time point the solution was replaced with a fresh collagenase Type II solution. The degradation percentage at each time point was defined as the ratio of the corresponding lost weight (*w*_0_ ‐ *w*_*ti*_) to initial weight (*w*_0_).

$$\%\;{\text{Degradation}}_{t=\mathrm{ti}}=\frac{w_{0}-w_{\mathrm{ti}}}{w_{0}}\cdot 100\%$$

### 3.7 Rheology test

Oscillatory rheology measurements were carried out on Anton Paar (MCR 302) by using a cone plate (radius 8 mm, cone angle 2°). A solvent trap was used to minimize water evaporation during the measurements. Temperature sweeps were performed from 5 to 40°C at a heating rate of 1°C/min. For all measurements a frequency of 1 Hz and a strain of 1% were applied. This strain and frequency were previously determined to be within the linear viscoelastic region of these polymer solutions.

### 3.8 In vitro adhesion tests

#### 3.8.1 Burst pressure test

The sealing capability of engineered sealants was measured according to a modified ASTM standard (F2392‐04) for burst pressure as described previously.^5^, ^11^ Briefly, 40 μl of prepolymer solution was injected and photocrosslinked on a 1 mm in diameter hole made on a collagen sheet. Air was pumped at a rate of 10 ml/min using a syringe pump and the pressure inside the seal was measured using a PASCO wireless pressure sensor and software until burst (Supp. Figure 1).

#### 3.8.2 Wound closure test

The adhesion strength of the engineered sealants was measured using a modified ASTM standard test (F2458‐05) according to previously published methods.^29^, ^30^ Porcine skin was used as the biological substrate in order to evaluate the relative adhesion strength of various formulations. The tissue was cut in 3 × 1 × 0.5 cm pieces and fixed onto two pieces of glass slides by superglue with a 0.5 cm overhang. Two opposing pieces were then placed next together and 100 μl of prepolymer solution was pipetted and photocrosslinked on 1 × 1 cm surface area via exposure to visible light (Supp. Figure 2). The adhesive strengths of the sealants were measured at the detachment point using an Instron 5542 mechanical tester. Tensile loading was conducted at strain rate of 1 mm/min. Adhesive strength was reported as the maximum stress on the stress–strain curve, corresponding to the breaking point.

#### 3.8.3 Lap shear test

The shear strength of the bioadhesives was measured using a modified lap shear test based on ASTM standard (F2255‐05) according to previously published protocol.^31^ As a biological substrate, porcine arteries (5 mm collapsed width) were cut into 20 mm long segments and fixed on glass slides by superglue. Prepolymer solution was then applied on half of one segment (10 × 5 mm), over which the second segment was placed (Supp. Figure 3). After photocrosslinking with visible light, the thickness of the hydrogel was measured with a digital caliper and the glass slides were loaded to Instron 5542 mechanical tester and pulled apart at a rate of 1 mm/min. Shear stress (kPa) was measured at the maximum stress where the two artery segments were separated using a BlueHill Universal software.

### 3.9 Ex vivo test using a porcine anastomosis model

Porcine carotid arteries were prepared by removal of the tunica adventitia. Segments with approximately 5 cm in length without branching were cut from parent vessel. To achieve the best outcome for sealing and anastomosis, we used a two‐step sealing procedure using two different formulations of the engineered glue: an elastic and soft formulation based on 15% GelMA/15% mELP composite, named Glue II, and a stiffer formation based on pure GelMA, 30% (w/v), named as Glue I. This procedure allowed for the penetration of the elastic glue (Glue II) without cracks to achieve a leak‐free anastomosis, and the subsequent reinforcement of the anastomosis site with the stiff glue (Glue I) to protect it against external stress.

In the first step of gluing, the composite prepolymer solution (Glue II) was injected directly in between two segments and crosslinked via exposure to visible light. Then, an anastomosis was made with an 18‐gauge needle from the donor artery to the recipient. In the second step of gluing, the GelMA prepolymer solution (Glue I) was injected on both sides of the interface between the two segments and crosslinked to strengthen the anastomosis site. To test the sealing, both ends of the recipient artery and one end of the donor artery were ligated with suture. The open end was connected to a syringe pump. Saline solution (0.9% NaCl in water) at 37°C was pumped at a rate of 4 ml/min and the pressure profile within the artery was measured using a PASCO wireless pressure sensor and Capstone software until the sealant failure. Different formulations of bioadhesives were tested in the two‐step procedure for anastomosis including the (i) 15% GelMA/15% mELP composite (elastic glue, Glue II) in Step 1 and the pure GelMA, 30% (w/v) (stiff glue, Glue I) in step 2; (ii) 15% GelMA/15% mELP composite (elastic glue, Glue II) in both steps; and (iii) pure GelMA, 30% (w/v) (stiff glue, Glue I) in both steps.

### 3.10 In vitro cell studies

#### 3.10.1 Cell line

HUVECs supplied from the American Type Culture Collection were cultured at 37°C and 5% CO_2_ in Lonza EBM Basal Medium (CC‐3121) with EGMTM Endothelial Cell Growth Medium Single Quots Supplements (CC‐4133). Cells were cultured in the flasks and passaged when reached the confluency of 70%.

#### 3.10.2 2D seeding on the composite bioadhesive

Glass slides were cut in 1 cm^2^ squares and coated with 3‐(trimethoxysilyl)propyl methacrylate to prevent hydrogel detachment during the in vitro studies. A total of 10 μl of prepolymer solution containing 15% GelMA/15% mELP was pipetted on a petri dish. The coated glass slides were placed over the drop of the prepolymer solutions with a 150 μm spacer and the hydrogel solution was crosslinked for 30 s by exposure to visible light. The glass slides containing crosslinked hydrogels were placed in a 24 well plate. Then, 40 μl of cell solution containing 10,000 cells (250,000 cell/ml‐media) was pipetted on the surface of each hydrogel, and incubated for 30 min at 37°C. After the initial incubation, 1 ml media was added to each well and the samples were further incubated for 7 days at 37°C in a 5% CO_2_ humidified atmosphere. The culture medium was replaced every 48 h.

#### 3.10.3 Assessment of cell viablity, proliferation, and spreading

A LIVE/DEAD™ Viability/Cytotoxicity Kit (Invitrogen) was used to determine the cell viability at days 1, 4, and 7. Ethidium homodimer (EthD‐1) and calcein AM were diluted into 50:1 and 200:1 with DPBS, respectively. The solution was then added to the cell‐seeded hydrogels and incubated for 45 min in 5% CO2 at 37 °C. Live and dead cells were observed by a fluorescence optical microscope (Primovert, Zeiss). Living cells were detected by calcein AM (green fluorescence), and death cells by EthD‐1 (red fluorescence). Then, the viability was obtained by calculating the number of viable cells divided by total number of viable and non‐viable cells using ImageJ (NIH) software.

A PrestoBlue (Invitrogen, P50200) assay was used to determine cellular proliferation. PrestoBlue is an inherently nonfluorescent dye that becomes fluorescent when reduced through redox reactions, such as when exposed to the reducing environments around proliferating cells. The level of fluorescence is thus used to quantify cell proliferation. A 10% v/v PrestoBlue solution was prepared in DPBS at dark. Media was removed from the previously seeded samples (*n* = 5) 24 h after seeding and 400 μl PrestoBlue solution was then added. The samples were then incubated at 37°C for 45 min to promote cell attachment. After incubation, the reduced PrestoBlue solutions were transferred to separate wells of a 96‐well plate and their fluorescence was measured with a plate reader (590 nm) on Days 1, 4, and 7 post‐seeding.

Cellular spreading on the composite hydrogel was assessed by Actin/DAPI and immunofluorescence staining. Cell‐seeded hydrogels were cultured under standard condition as described before. After 7 days, the samples were fixed for 1 h at room temperature using 4% v/v paraformaldehyde (Sigma‐Aldrich) in DPBS. Cells were permeabilized by soaking the samples in 0.1% v/v Triton X‐100 (Sigma‐Aldrich) dissolved in DPBS for 30 min while non‐specific binding was inhibited using 10% v/v bovine serum albumin (BSA, Sigma‐Aldrich) for 1 h at room temperature. Samples were then incubated for overnight at 4 °C in a solution containing the primary antibody (rabbit polyclonal anti‐CD31 (ab28364, Abcam)) at 1:200 dilution in 10% v/v BSA and 0.1% v/v Triton X‐100 in DPBS. Samples were then incubated for 1 h at room temperature in a solution containing secondary antibody at 1:400 dilution in 10% v/v BSA in DPBS. For Actin staining, cells were incubated with Alexa Fluor 488‐phalloidin in 0.1% w/v BSA at 37 °C (1:200 dilution) for 45 min to stain the actin cytoskeleton. Nuclei of the cells were also stained by 40,6‐diamidino‐2‐phenylindole (DAPI, Invitrogen). Images were taken from the samples using a fluorescence optical microscope (Primovert, Zeiss).

### 3.11 In vivo studies

#### 3.11.1 Subcutaneous implantation

A rat subcutaneous implantation model was used to assess the in vivo biodegradation and biocompatibility of the engineered bioadhesives. All animal experiments were reviewed and approved by Institutional Animal Care and Use Committee (ICAUC; protocol #2018‐076) at UCLA (Los Angeles, CA). Male Wistar rats (200–250 g) were put under general anesthesia (isoflurane, 2.5%). After inducing anesthesia, eight (8 mm) incisions were made on the posterior dorsomedial skin and small lateral subcutaneous pockets were prepared by blunt dissection. Cylindrical hydrogel samples were weighed, lyophilized for 24 h, and reweighed. Afterward, the lyophilized samples were sterilized via exposure to UV light for 10 min. The samples were subcutaneously implanted in rats. The animals carrying the hydrogel implants were euthanized on Days 7, 28, and 56 post‐surgery. The hydrogel samples were explanted and used for histopathological or degradation analyses.

#### 3.11.2 In vivo biodegradation profile

The samples (*n* = 3) were carefully cleaned to remove any surrounding tissue and weighed (*w*_0_). The in vivo degradation profile was then obtained similarly to the in vitro counterpart. The degradation percentage at each time point was defined as the ratio of the corresponding lost weight (*w*_0_ *‐ w*_*ti*_) to initial weight (*w*_0_).

#### 3.11.3 Histological analysis and immunofluorescent staining

The samples with intact tissue were fixed in 4% v/v paraformaldehyde solution in DPBS for 4 h at 4°C. After fixation, they were soaked first in 10% w/v sucrose solution in DPBS for 4 h, then in 30% w/v sucrose solution in DPBS for 24 h at 4°C. The samples were then flash‐frozen in O.C.T. compound (Fisher) using liquid nitrogen. The blocks were sectioned into 10 μm thick slices using a cryostat (Leica CM1950). Samples from both groups were used for (I) hematoxylin/eosin (*n* = 3), (II) CD3/DAPI (*n* = 3), and (III) CD68/DAPI (*n* = 3) staining.^32^ Anti‐CD3 antibody (ab5690 ‐ Abcam) and Anti‐CD68 antibody (ab125212 ‐ Abcam) were used as primary antibodies with AlexaFluor 488 goat anti‐rabbit (IgG) secondary antibody (ab150077) in accordance with the manufacturer protocols. The samples were then imaged using an inverted fluorescence microscope (Zeiss Axio Observer Z7).

### 3.12 Pig nonsurvival anastomosis model

A newly developed in vivo swine model was used to assess the feasibility of applying our two‐step sealing procedure for the endovascular anastomosis. The endovascular bypass was performed in swine (50–140 lbs, *n* = 4) under general anesthesia. The right common carotid and ascending cervical arteries were then surgically exposed, cleaned of adventitia, and loosely looped with 3–0 silk through a para‐midline linear skin incision. Topical papaverine hydrochloride was applied to the exposed arteries for vasodilation. The two arteries were placed closely in parallel and gently unmoistened with gauze. The composite bioadhesive prepolymer solution (15% GelMA/15% mELP) was applied directly to the arteries and crosslinked with visible light for 160 s. Then, the pure GelMA bioadhesive prepolymer solution (30% w/v GelMA) was applied over the already crosslinked composite gel and crosslinked with visible light for 160 s. Percutaneous arterial access was then obtained by femoral artery puncture. Then, 100 IU/kg heparin was administered IV. Then, the femoral artery sheath was entered with an Outback reentry catheter (Cordis, Bridgewater, NJ), which was advanced to the carotid artery under fluoroscopic guidance. The Outback reentry needle was deployed through the walls of both the carotid and ascending cervical arteries and a 0.014‐in. Transcend guidewire (Stryker Neurovascular, Fremont, CA) advanced coaxially into the recipient vessel. The anastomosis was subsequently dilated with a Gateway angioplasty balloon and finally a Wingspan stent (Stryker Neurovascular) was placed.

### 3.13 Statistical analysis

Unpaired, one‐tailed Welch's *t* test was applied with a 95% confidence interval with a minimum of three replicates per sample. Error bars represent mean ± SD of measurements (*p < .05, **p < .01, and ***p < .001).

## 4 CONCLUSION

In this work, we introduced a new class of composite hydrogel sealants primarily for a novel, arterial anastomosis procedure, which can be fine‐tuned to be utilized for a broader range of vascular applications. Composite hydrogels were fabricated from two biopolymers, mELP and GelMA. The synergistic association of these two biopolymers with distinct physicochemical properties enabled fine‐tuning of various properties of the formulated glues including mechanical properties, in vitro and in vivo degradation, deswelling, and adhesion. The composite GelMA/mELP hydrogel was shown to support the growth, spreading, and proliferation of HUVECs in vitro. Furthermore, GelMA/mELP composite hydrogel elicited a short‐lived inflammatory response and was shown to be efficiently biodegraded in vivo when implanted subcutaneously in a murine animal model. Taken together, our results demonstrate the remarkable potential of GelMA/mELP composite hydrogels for vascular applications, in particular for the proposed endovascular anastomosis procedure. However, for clinical application purposes, additional studies are recommended to optimize the (i) size of the anastomosis according to the predetermined parameters unique to each patient and (ii) rate of glue degradation relative to tissue healing. The former is to ensure sufficient blood flow to both arteries; the latter is critical to maintain the structural integrity of the glue, securing the anastomosis.

## AUTHOR CONTRIBUTIONS

**Gokberk Unal**: Conceptualization; data curation; formal analysis; investigation; methodology; validation; writing‐original draft; writing‐review and editing. **Jesse Jones**: Conceptualization; data curation; formal analysis; investigation; methodology; validation; writing‐original draft; writing‐review and editing. **Sevana Baghdasarian**: Data curation; formal analysis; investigation; methodology; writing‐review and editing. **Naoki Kaneko**: Conceptualization; data curation; formal analysis; investigation; methodology; validation; writing‐original draft; writing‐review and editing. **Ehsan Shirzaei Sani**: Data curation; formal analysis; investigation; methodology. **Sohyung Lee**: Data curation; formal analysis; investigation; methodology. **Shima Gholizadeh**: Data curation; formal analysis; investigation; methodology; writing‐review and editing. **Satoshi Tateshima**: Conceptualization; methodology; resources; supervision; writing‐review and editing. **Nasim Annabi:** Conceptualization; funding acquisition; investigation; methodology; resources; supervision; validation; writing‐original draft; writing‐review and editing.

5

### PEER REVIEW

The peer review history for this article is available at https://publons.com/publon/10.1002/btm2.10240.

## Supporting information

**Appendix S1** supporting information
