# Supplementary material for: Engineering elastic sealants based on gelatin and elastin‐like polypeptides for endovascular anastomosis
Source: Bioeng Transl Med. 2021 Aug 10;6(3):e10240. doi: 10.1002/btm2.10240 (PMC8459633; doi:10.1002/btm2.10240)
Supplement: Supplementary file 1 — Appendix S1 supporting information [file BTM2-6-e10240-s001.docx]

**Supplementary Information**

**
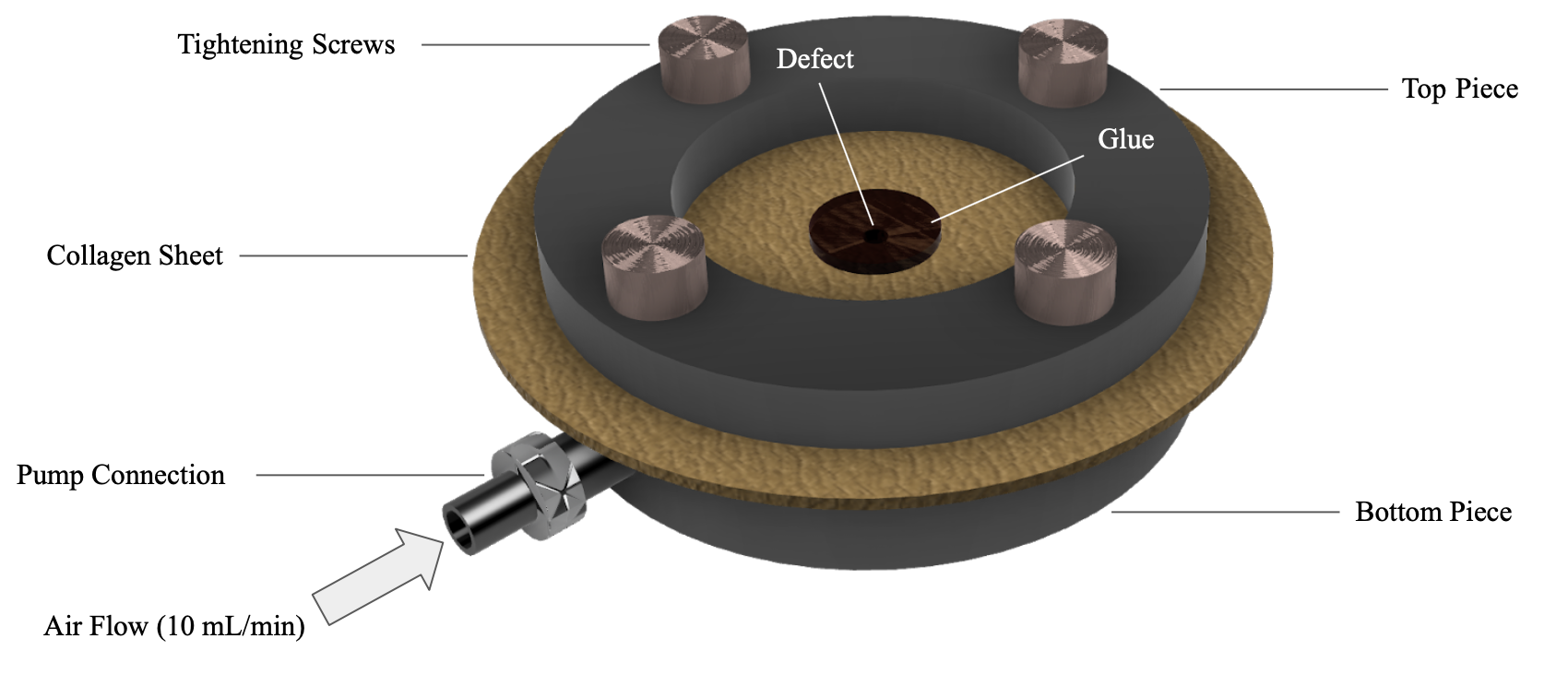
**

**Figure 1.** Experimental set up used for the burst pressure test.

**
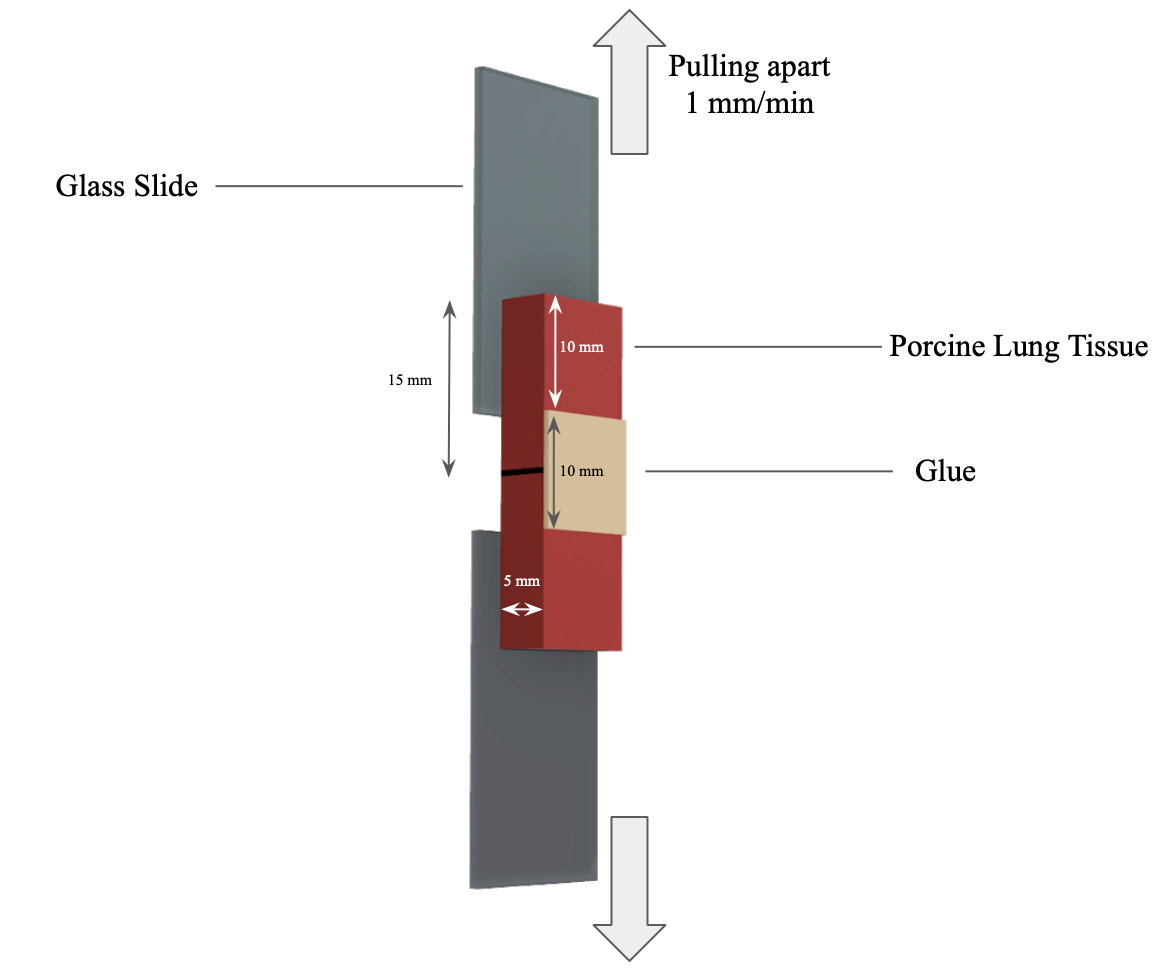
**

**Figure 2.** Experimental set up used for the wound closure test.

**
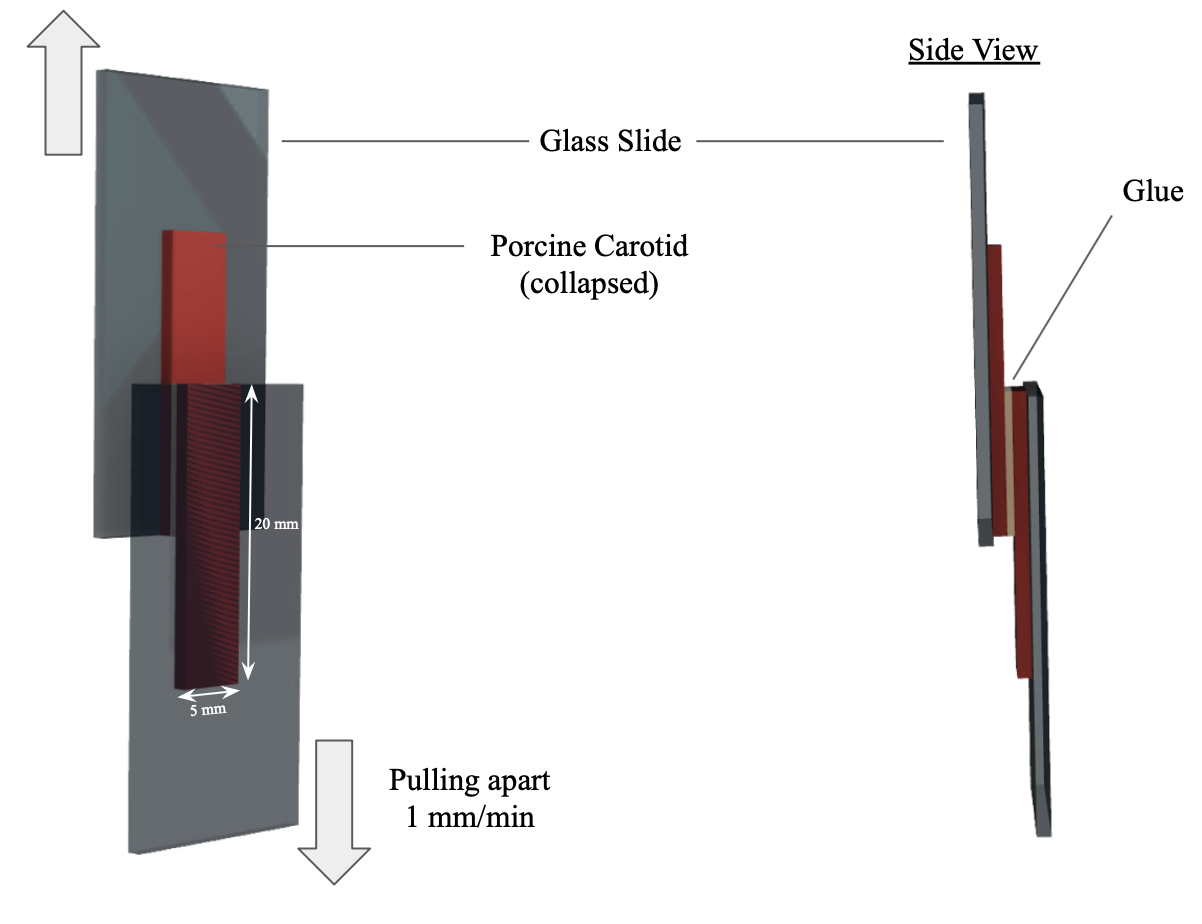
**

**Figure 3.** Experimental set up used for the lap shear test.


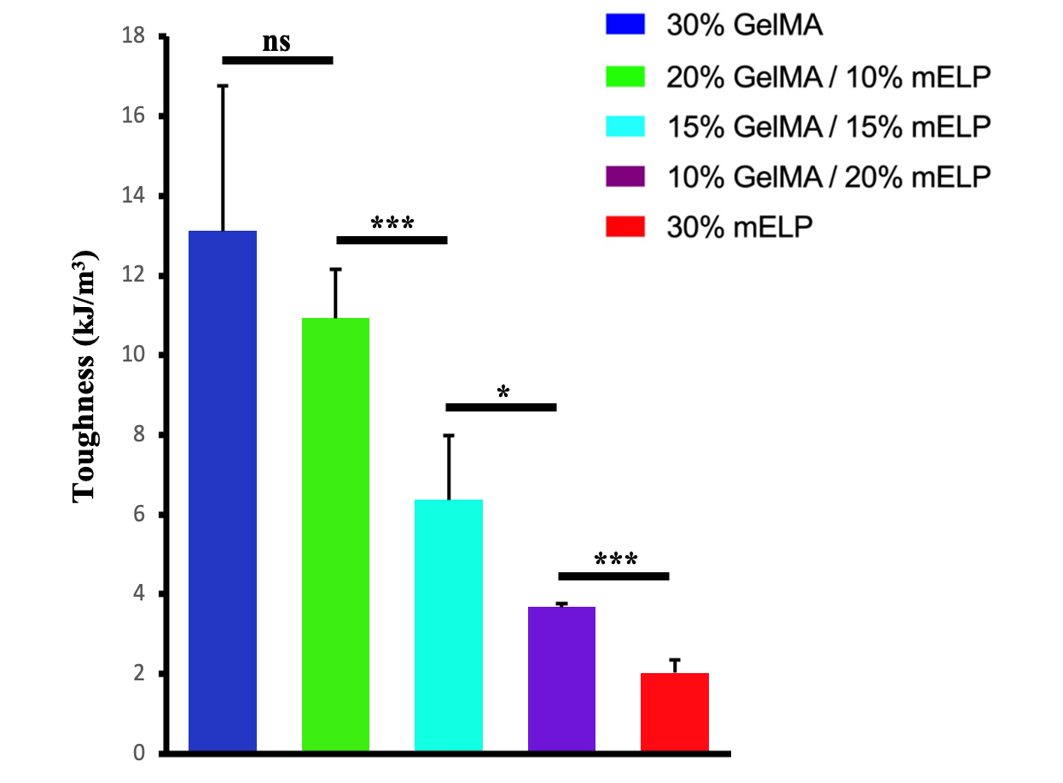


**Figure 4.** Toughness values for hydrogel formulations made of various concentrations of mELP and GelMA obtained during the first loading-unloading compression cyclic test without interval under a constant strain (ɛ = 40%) (* p < 0.05, *** p < 0.001).


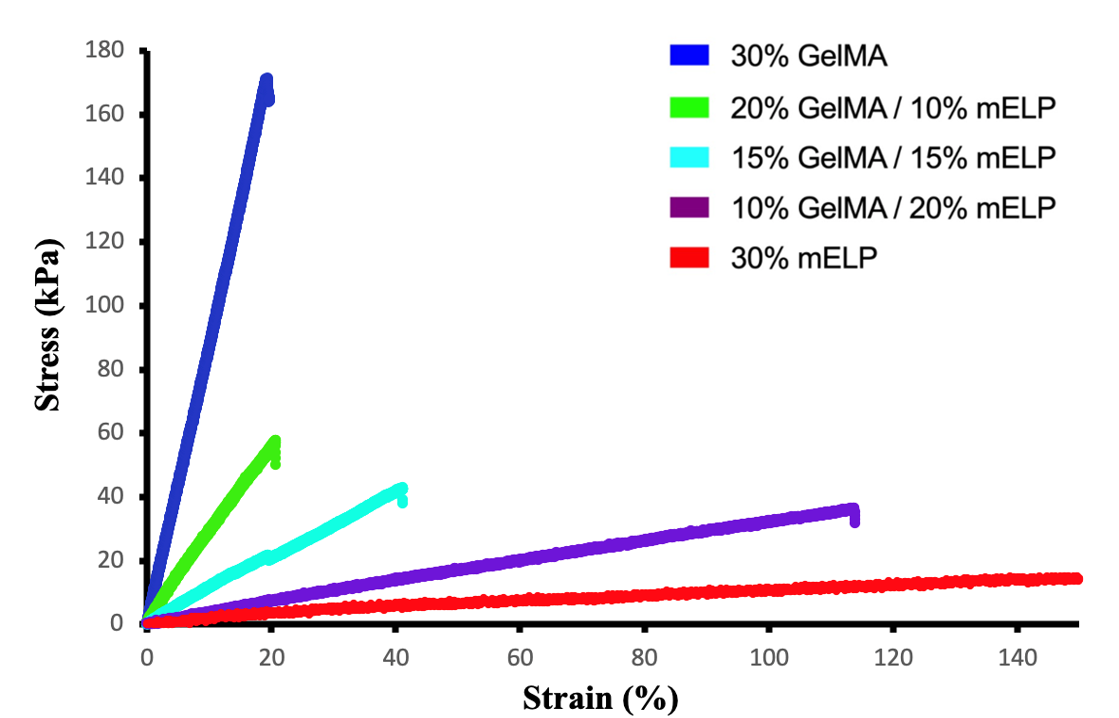


**Figure 5.** The representative stress vs strain curves for hydrogel formulations made of various concentrations of mELP and GelMA based on tensile testing.
